# Parental Use and Educational Campaigns on Sunbed Use Among Teenagers and Adolescents

**DOI:** 10.1097/MD.0000000000003034

**Published:** 2016-03-18

**Authors:** Ignazio Stanganelli, Luigi Naldi, Fabio Falcini, Serena Magi, Laura Mazzoni, Matelda. Medri, Rita Bertoncini, Ombretta Calderoni, Veronica Agnoletti, Luca Nadiani, Giuseppe Palmieri, Sergio Di Nuzzo, Calogero Pagliarello, Sara Gandini

**Affiliations:** From the Skin Cancer Unit (IS, SM, LM, MM), Istituto Scientifico Romagnolo per lo Studio e la Cura dei Tumori (IRST) IRCCS, Meldola; Centro Studi Gruppo Italiano Studi Epidemiologici in Dermatologia—Fondazione per la Ricerca (LN), Ospedale Maggiore Presidio Ospedaliero Matteo Rota, Bergamo; Romagna Cancer Registry (FF), Istituto Scientifico Romagnolo per lo Studio e la Cura dei Tumori (IRST) IRCCS, Meldola; Istituto Oncologico Romagnolo (RB, LN) (IOR), Forli; Dermatology Unit (OC), Azienda Unità Sanitaria Locale della Romagna, Ravenna; Centro Studi Avanzati sull’Umanizzazione delle Cure e sulla Salute sociale (Ce.Um.S.) (VA), Sociology Department, University of Bologna; Unit of Cancer Genetics (GP), Institute of Biomolecular Chemistry, National Research Council (CNR), Sassari; Clinica dermatologica (SDN, CP), University of Parma; and Division of Epidemiology and Biostatistics (SG), European Institute of Oncology, Milan, Italy.

## Abstract

This study aimed to evaluate the prevalence of sunbed use among teenagers and the association between familial behavior and the adoption of UV-protective practices in this age group. We also assessed the impact of an educational program on students’ knowledge about the potential risks of sunbed use. The educational intervention focused on: (i) skin effects of UV radiation, (ii) photoaging and photocarcinogenesis, (iii) risk factors for skin cancer, (iv) indoor sun tanning and misleading concepts such as possible protective effect of sunbed use on skin cancer risk, (v) sun protection and relation with skin phototype, and (vi) early diagnosis of melanoma using the ABCDE check list and the ugly duckling sign. We carried out a survey of 3098 students and found a strong association between parental sunbed use and students’ use of the same (*P* < 0.0001). Students who attended the educational intervention were more aware that sunbed use cannot prevent sunburns (*P* = 0.03) than those who did not attend, making adjustments for confounding variables. However, sunbed use by parents influenced the desire to use a sunbed more than participation in the educational intervention (*P* < 0.0001). In conclusion, although our results indicate that educational interventions can improve knowledge of the risk of sunbed use. They also reveal a strong correlation between sunbed use by teenagers and parental behavior that highlights the importance of educational interventions involving families.

## INTRODUCTION

Cutaneous melanoma and nonmelanoma skin cancer (NMSC) have undergone a substantial increase in incidence in developed countries over the last few decades^1,2^ and are susceptible to early preventive measures.^3^ Ultraviolet light (UV) exposure is an established risk factor for cutaneous melanoma and NMSC^4,5^ and primary prevention is a crucial to decreasing the number of malignant skin cancers and costly melanoma treatments. In 2009, the International Agency for Research on Cancer classified the entire UV spectrum as carcinogenic.^5^ The pattern of sun exposure may affect the risk of different skin cancers and the risk of the same cancer in different body locations in different ways.^6,7^ Recent studies have highlighted an increasing incidence of cutaneous melanoma and NMSC among young adults in several countries.^8–10^ Although the lifetime risk of melanoma is higher in men than in women, recent studies have documented that the opposite is true for young adults and adolescents, where the female–male incidence ratio is as high as 1.8.^9^ This finding may be attributable to some sex-specific behavior that lead to different UV light exposure.^11^ A meta-analysis reported that sunbed use before the age of 35 years substantially increases the risk of melanoma.^12^ Over the last few decades, indoor tanning has become increasingly popular among adolescents, especially girls.^13^ However, Italy, like many other countries in Europe, have further increased nationwide restrictions on the use of indoor tanning by people under 18.^14,15^ Melanoma is significantly associated with sun and sunbed exposure but the risk is modified by several host factors. A systematic meta-analysis of all studies published from 1996 to 2002 showed that the most important phenotypical factors for melanoma are hair, eye and skin color, skin type, presence of freckles, and number of common nevi.^6^

There is widespread consensus that UV protection habits should begin early in life and be taught as part of routine preventive healthcare.^16,17^ A systematic review^18^ concluded that education approaches to increasing UV-protective behavior were effective when implemented in primary schools and in recreational settings and that insufficient evidence was available when implemented in other settings.

“‘SAVE THE SKIN” was a complex project funded by a nonprofit association called *Istituto Oncologico Romagnolo* (*IOR*) and including educational and research components.^19,20^ As a combined research-intervention project, the “SAVE THE SKIN” project served both educational and survey purposes. The survey questions covered areas of social medicine, preventive dermatology, and epidemiology of skin cancer risk factors. The general objective was to assess prevalence of sunbed use among teenagers and the association between familial behavior and the adoption of UV-protective practices in youth. A pilot educational intervention was also conducted among students to determine the impact of an educational programm on awareness of this issue.

## SUBJECTS AND METHODS

The “SAVE THE SKIN” project was developed as part of the IOR Melanoma Project, a clinical core integrated into the Skin Cancer Unit of *Istituto Scientifico Romagnolo per lo Studio e la Cura dei Tumori* (*IRST IRCCS*) with the partnership of the Centre for Advanced Studies on the Humanization of Care, Health and Social Safety, University of Bologna and the European Institute of Oncology in Milan. The project included a summer beach survey^19,20^ and a workshop for beauty salon and sun tanning centre operators aimed to highlight the risk of artificial radiations and to provide information from the Italian Ministerial Decree of May 2011 on the limitation of artificial UV devices in minors and in a high-risk subgroup of the population.^14^ Furthermore, a specific project module consisted in a survey combined with the piloting of an educational intervention in secondary schools of Ravenna and Forlì, 2 towns in east-central Italy.^19,20^

This study was approved by the Institutional Review Board (March 2011) of the “Centro Studi GISED” coordinating the Italian Group for Epidemiologic Research in Dermatology aimed at improving scientific knowledge and research into dermatological diseases. Written informed consent was obtained from each participant's next of kin and the procedure was approved by Ethics Committee. Eight secondary schools (6 in Ravenna and 2 in Forlì) participated in this project. All the students (ages ranging from 13 to 20 years) were invited to take part in the survey. A subsample of 9 classes from 6 schools in Ravenna was also drawn randomly to participate in an Educational Intervention (EI). Students were between the ages of 14 and 19.

The EI was planned according to the Italian National Cancer Recommendations for 2010 to 2012, which included guidelines on activities for primary and secondary prevention of skin cancer. It should be noted that along the Romagna coastline, sunbathing on the beach is one of the most popular recreational activities among young people.^19,20^

The EI was conducted between November and December 2011 in school meeting halls and consisted of an interactive 2-hour meeting with students and teachers focusing on: (i) skin effects of UV radiation, (ii) photoaging and photocarcinogenesis, (iii) risk factors for skin cancer, (iv) indoor sun tanning and misleading concepts such as possible protective effect of sunbed use on skin cancer risk, (v) sun protection and relation with skin phototype, and (vi) early diagnosis of melanoma using the ABCDE check list and the ugly duckling sign. Lessons were held by an expert dermatologist (OC) or a specifically trained biologist (SM) using an identical didactic multimedia presentation. After the training lesson, education resource material was distributed. In January to March 2012, additional activities aimed at the production of educational materials were carried out in the student classes that received training. During the primary prevention EI, students used different techniques to produce drawings, videos, and graphic projects that illustrated the correct behavior for adequate sun protection, the hazards of excess UV radiation, and the benefits and harmful effects of UV radiation. This process was monitored by teaching staff and by 2 of our team (SM, RB). Furthermore, to promote active participation by the students in the educational program, *IOR* project funding launched an award for the winner of the best presentation highlighting the importance of primary skin cancer prevention.

**FIGURE 1:**
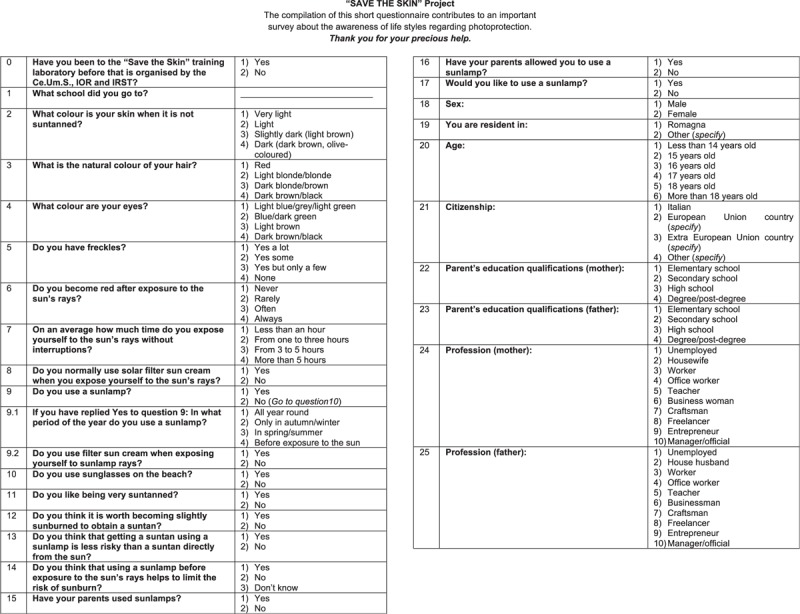
Questionnaire.

The survey, based on a paper questionnaire, was conducted between April and May 2012 (6 months after the main educational intervention and 2 months after the end of additional activities) and involved all the students of the secondary schools, including those who had not participated in the educational intervention. The questionnaire consisted of 25 questions (Figure 1) and was developed on the basis of our previous experience with similar surveys^21^ and on published studies about sun protective behavior in children and adolescents.^22^ The questionnaire explored sun exposure habits, attitudes to the use of sunbeds, self-reported sunburn history, and sun protection behavior. Information on skin phenotype, socioeconomic variables, and parental role on sun protection was also collected. Copies of the questionnaire were distributed systematically on a daily basis during school hours and were collected at the school desk. Teachers and one of the co-authors (RB) distributed the paper questionnaires and asked the students to complete them by hand and return them to the school desk. Around 10 to 15 minutes were needed to fill in the questionnaire and a total of 3098 questionnaires were completed (98% response rate).

### Statistical Methods

Descriptive analyses were carried out on data derived from the questionnaire. Data were stratified on the basis of (1) the response ‘have used sunbeds’ versus (vs) ‘have never used sunbeds’ and (2) the participation of the students in the educational intervention. Differences between frequencies were assessed using chi-square tests for independence of categorical variables and by logistic regression including all significant factors and confounders. The role of factors influencing sunbed use, the desire to use them and the opinion about their safety was assessed by calculating the odds ratios and 95% confidence interval (95%CI) derived from multiple logistic regression analysis for selected predictive variables. Response variables of the logistic regression models were “have used sunbeds,” the desire to use a sunbed and the awareness about the safety of sunbed use (“Is sunbed less risky than sun exposure?”). We also evaluated the efficacy of the EI including a categorical variable (“participation vs non-participation”) in the multivariate logistic regression model. Only factors significantly associated with the response variables were maintained in the models.

## RESULTS

### Sunbed Use

The vast majority of students were resident in Romagna (n = 2872, 93%) and were <18 years of age (n = 2055, 66%). About 2% were <14 years old, 25% were 15, 20% were 16, 20% were 17, 20% were 18, and 12% were ≥19. About 8% (n = 229) attended the educational intervention.

Information provided by students on their use of sunbeds and on parental characteristics and behavior is reported in Table 1. The prevalence of sunbed use among students was 7% (n = 216).

**TABLE 1:**
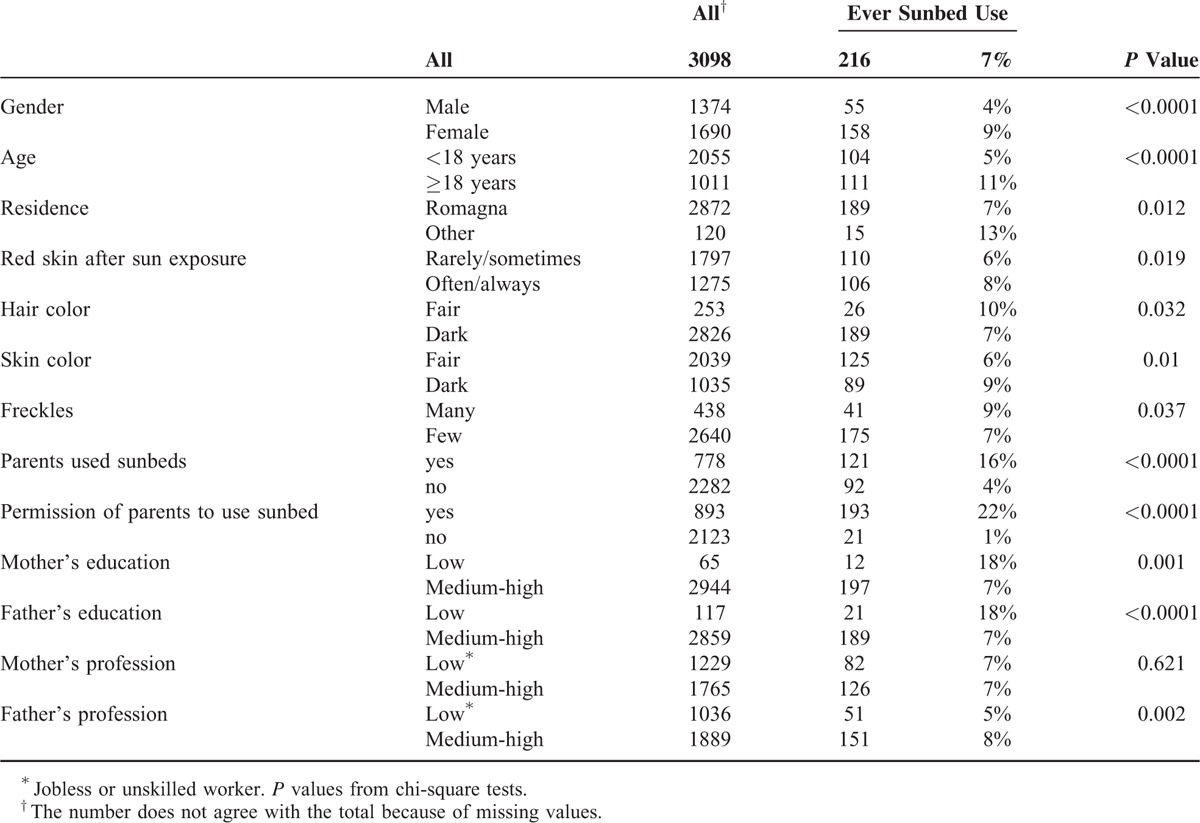
Prevalence of Sunbed Use According to the Characteristics of Adolescents and Parents

More females than males reported using sunbeds (9% vs 4%; *P* < 0.0001) and teenagers <18 years reported using sunbeds less frequently than those aged ≥18 (5% vs 11% for age<18 and age≥18; *P* < 0.0001). Furthermore, subjects with risk phenotypes used sunbeds more than others. Students who often/always experienced red skin after sun exposure used sunbeds more frequently (8% vs 6%; *P* = 0.02). Students with light hair color used sunbeds more frequently than those with other hair colors (10% vs 7%; *P* = 0.03). Subjects with numerous freckles used sunbeds more frequently than those with few freckles (9% vs 7%; *P* = 0.04). Fair skin color was the only phenotypical factor associated with lower sunbed use: 6% of students with fair skin color use sunbeds versus 9% of students with dark skin color (*P* = 0.01).

The prevalence of sunbed use was 25% (n = 778) among parents. Almost a third of students declared have had their parents’ permission to use the sunbed (n = 893, 29%).

If parents used sunbeds, their children were more likely to report using sunbeds as well (16% vs 4% for parents’ use vs no use; *P* < 0.0001).

Parents’ levels of education were very important in influencing the use of sunbeds by students. The proportion of students using sunbeds was significantly higher if parents had only had an elementary school education compared to those with a higher educational level (18% vs 7% for low vs high educational level of mother and father: *P* < 0.001). We assessed Socio-Economic Status (SES), considering parents’ professions (unskilled vs skilled workers). Low SES, indicated by the level of the father's profession was associated with limited sunbed use by the students (5% vs 8% for unskilled and skilled workers, respectively; *P* = 0.002). Conversely, evaluating parents’ educational levels we observed that a medium–high educational level was associated with a significantly lower sunbed use than that seen for low educational level parents (Table 1).

Multivariate logistic models (Table 2) show that sunbed use by parents and parental permission was significantly associated with sunbed use, after adjusting for confounding variables such as gender, parental education, and profession.

**TABLE 2:**
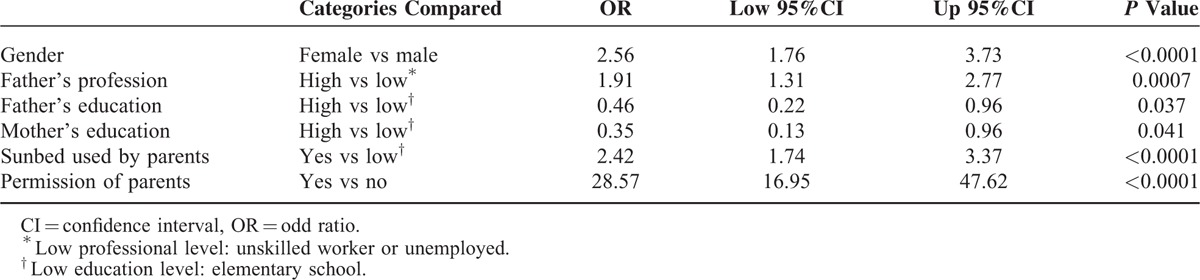
Multivariate Logistic Models Evaluating Factors Associated With “Ever Sunbed Use”

### Educational Intervention

Overall, 229 students participated in the EI. Data on students’ features, risk factors, and awareness of melanoma risk by participation in the EI on melanoma prevention are reported in Tables 3 and 4. Some differences were found in the 2 student group, that is, in the EI group there were significantly more girls (*P* = 0.0002), older students (*P* < 0.0001), and a higher number of students with fair hair color (*P* = 0.02).

**TABLE 3:**
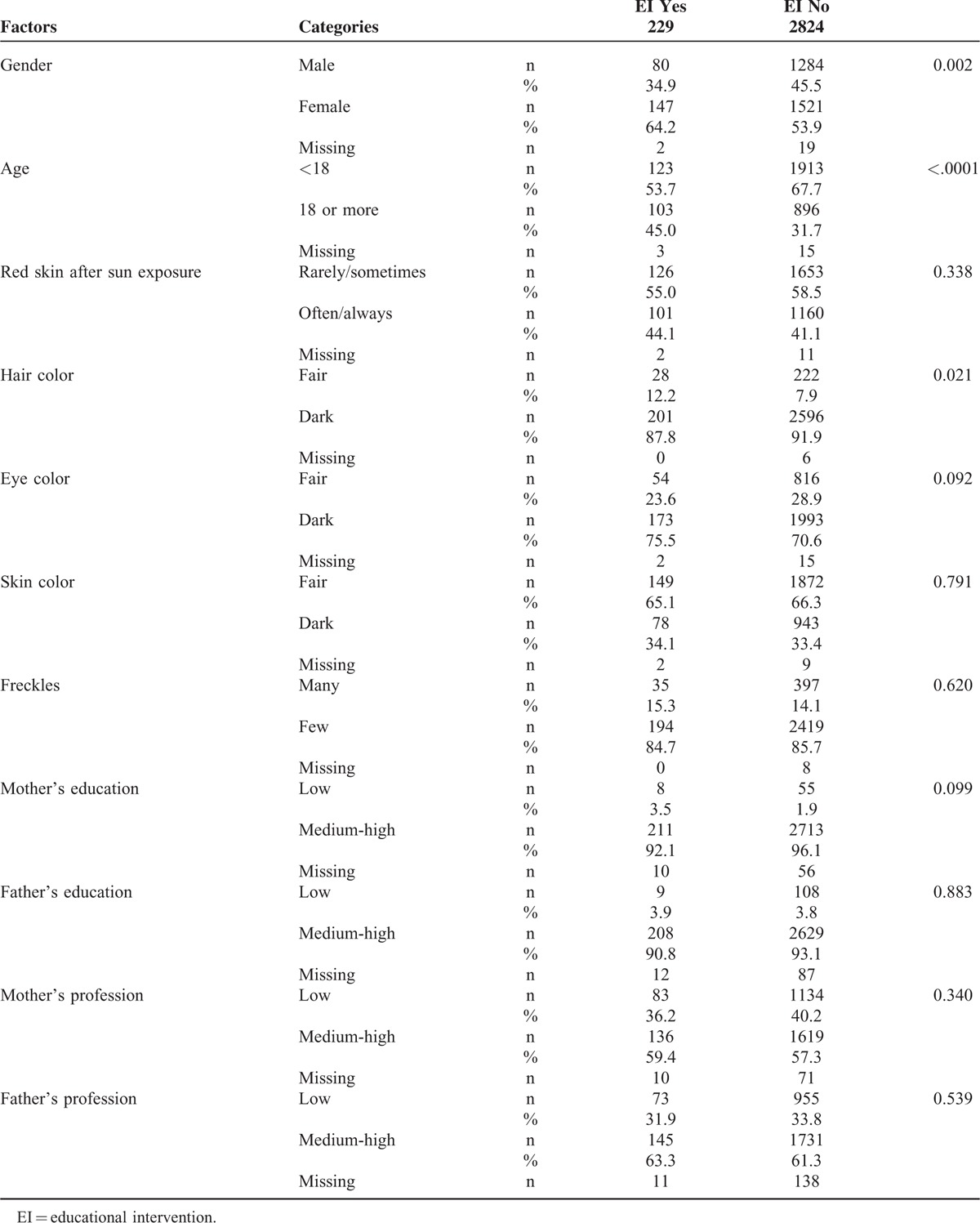
Student Characteristics on the Basis of Participation in EI on Melanoma Prevention

**TABLE 4:**
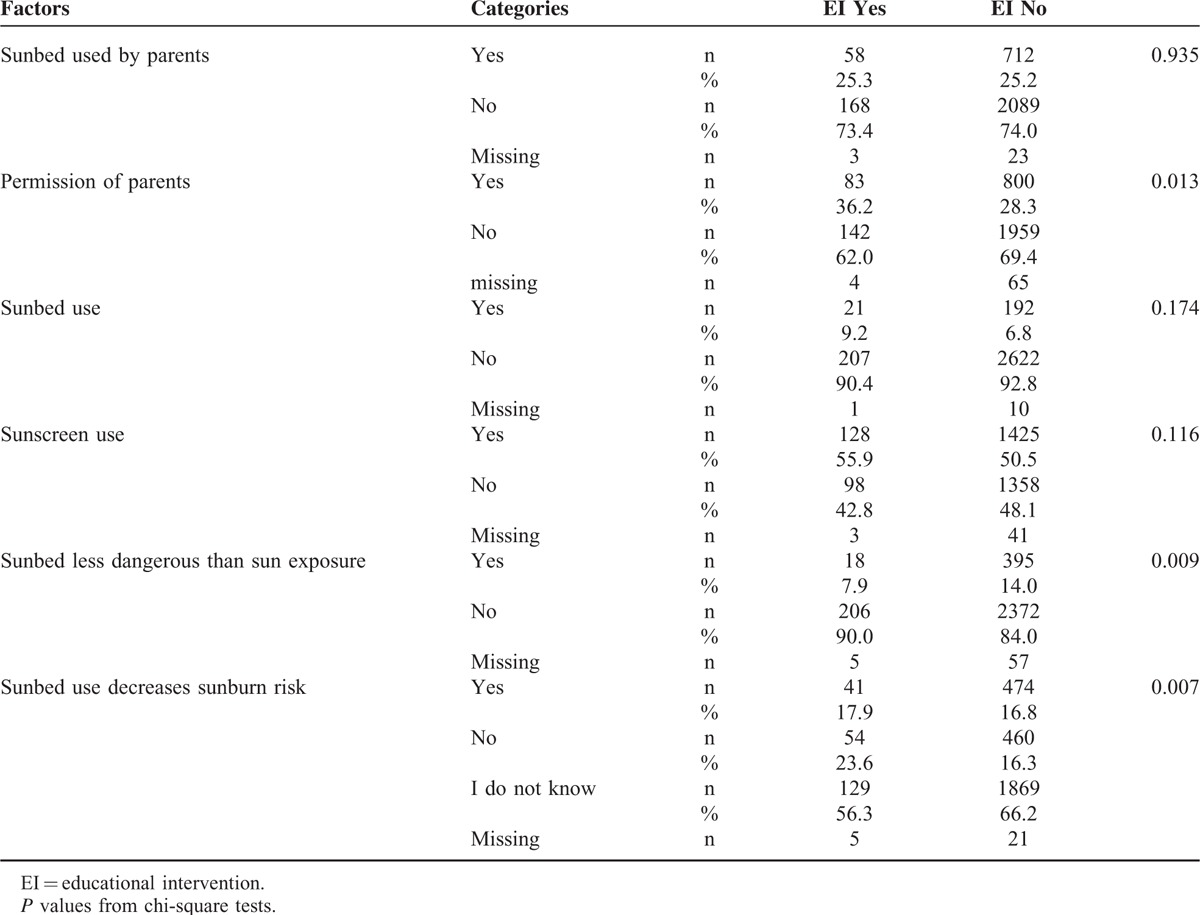
Risk Factors and Knowledge by the Participation in EI on Melanoma Prevention

No difference was seen in the use of sunbeds between students who attended the EI and those who did not (9% vs 7% respectively; *P* = 0.17). However, students who attended the EI were more aware of the risks. The proportion of students who believed that sunbed use was less risky than sun exposure was significantly higher in the group who did not attend the EI (14 vs 8 %; *P* = 0.009). Although EI students were also more aware that sunbeds cannot prevent sunburns (24% vs 16%, for the education group vs others, respectively; *P* = 0.007), more than half of the students in the group (56%) did not know whether the use of sunbeds could decrease sunburn risk.

Multivariate logistic models show that the use of sunbeds by parents was more important (*P* < 0.0001) than EI participation in influencing the desire of students to use sunbeds, making adjustments for confounding variables such as gender, parental profession, and skin type (Table 5). However, it is of note that EI participation significantly improved (*P* = 0.03) the knowledge that sunbed use is not less risky than sun exposure, adjusting for other confounding variables.

**TABLE 5:**
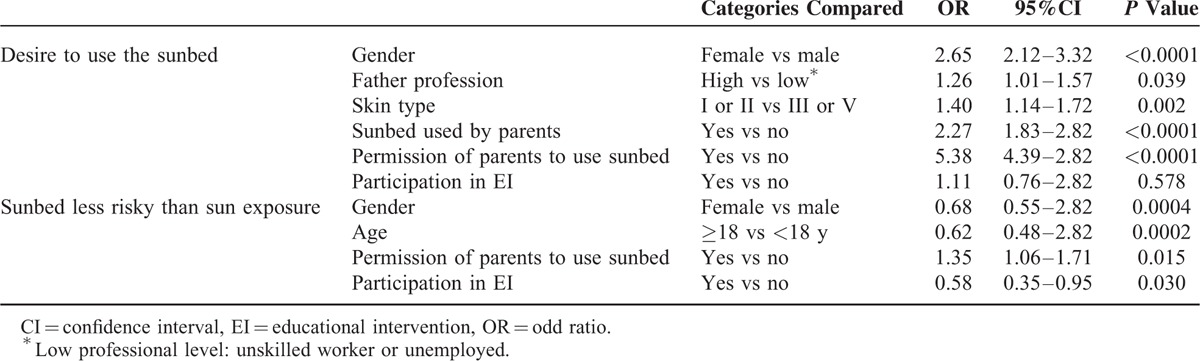
Results From Multivariate Logistic Models

## DISCUSSION

This study was aimed at evaluating the association between sunbed use and the adoption of sun-protective practices in young people with familial behavior. We also assessed the impact of an educational program on students’ knowledge about melanoma risk due to sunbed use.

Our survey reveals that ∼7% of adolescents in the Romagna region use sunbeds and that the use of sunbeds is associated with several individual characteristics, including gender, skin phenotype, and socioeconomic status. Fair phenotype, female gender and lower SES of parents are associated with a higher sunbed use. More importantly, our data reveal a strong correlation between sunbed use by parents and their use by children, indicating the importance of educational interventions involving families. Finally, our study highlights that, although a brief educational intervention may improve knowledge and attitudes, we do not know whether it can change habits. Our survey was a pilot study and future research will take into consideration these preliminary data.

Our estimates of sunbed use by adolescents were lower than those reported in a systematic review of data from the United States, Europe, and Australia, pointing to a prevalence of exposure of 35.7% (95% CI, 27.5–44.0%) in adults, 55.0% (33.0–77.1%) in university students, and 19.3% (14.7–24.0%) in adolescents.^13^ This last percentage may be a consequence of a law passed in Italy prohibiting sunbed use by under 18 seconds. However, a German study showed that the prevalence of sunbed use in minors is very high, despite a legal ban in this age group, thus underlining the importance of education.^23^ With regard to predictive variables of exposure, our data are generally in agreement with those of previous surveys^24–26^ showing that sunbed use is influenced by several psychosocial and demographic variables including being female, being a teenager, having more pocket money, having a parent who uses an indoor tanning bed and who allows their adolescent child to use it, and holding certain beliefs about the consequences of indoor tanning.

Young people state that they primarily use sunbeds to get a suntan and to appear more attractive.^27^ All these variables should be taken into account when planning educational interventions aimed at reducing sunbed use.^28^ Our preliminary survey indicates that an educational intervention designed to reduce UV light exposure increases short-term awareness.

It has been seen that sun protection programs are generally more successful in improving sun-protective practices for infants (by parents) and among younger children, but less successful among adolescents.^29^ Efforts in sun-protection education, supportive environments, and policies are difficult to sustain effectively as primary school children transfer to secondary schools.^30^ Interventions in secondary schools are potentially important because adolescents and young adults are more likely to be exposed to UV radiation than younger children and are less likely to adopt sun-protective behavior. Parents and caregivers have less influence in promoting sun protection in this age group and high schools and colleges can provide an infrastructure to support intervention activities.^18^ Despite high levels of knowledge about the health effects of unprotected sun exposure, changes in attitudes and social norms during adolescence are associated with an increase in high-risk behavior and present a unique challenge to health educators.^31^ Interestingly in our survey, in agreement with previous studies, adolescents who reported using sun protection most of the time were the least likely to report the use of an indoor tanning bed.^32^ Moreover, the probability of using sunbeds was much greater among adolescents whose parents also used indoor tanning than in those whose parents did not. The same result was reported in other studies, pointing to the importance of social influences and perceived norms on behavior.^33,34^ Our results showed that sunbed use in adolescents was associated with lower educational levels of parents but also higher professional levels of parents. Hence, education and profession that are 2 aspects related to socio-economic factors seem to have different effects: education of parents seem to have a stronger effect on awareness than professional levels, which is a better proxy of socio-economical status.

Furthermore, previous studies have shown that known risk groups, that is, individuals with a history of sunburn, pigmentation marks and familial melanoma history, do not use solariums significantly less than those who are risk-free.^34^ Our findings confirm that students with a high-risk phenotype (skin becomes quickly red after exposure) use a sunbed significantly more than others, which is alarming. It is interesting to note that the fair phenotype is associated with less sunbed use than the dark phenotype, whereas a history of sunburns after sun exposure is associated with a higher incidence sunbed use (probably because the sunbed is used as a means to protect the skin from the risk of sunburn before intense sun exposure).

We are aware that our study had limitations. We used a regional sample of secondary school students who may not be representative of the general Italian population of the same age group. It would be useful to reproduce these findings in other regions. Other limitations were self-reporting, lack of data on the frequency of exposure to use of sunbeds, and the fact that no information on family-owned sunbeds was collected. Furthermore, the questionnaire we used is similar to others used in the literature, but has never been validated.^21,22^ In the present study it was not possible to assess the long-term impact of the educational intervention, but we are now planning to use pre- and postintervention questionnaire to evaluate whether this type of intervention with students can also help in changing habits. Despite these limitations, our data indicate that young people are exposed to sunbeds to a substantial degree and that exposure is influenced by potentially modifiable factors.

In conclusion, our data indicate a high proportion of exposure to sunbed use among adolescents in Italy and highlight the existence of multiple determinants of behavior, pointing to the need for collaboration across multiple sectors to develop educational interventions aimed at reducing indoor tanning and preventing future cases of skin cancer.^35^

## References

[1] de Vries E Coebergh JW Cutaneous malignant melanoma in Europe. *Eur J Cancer* 2004; 40:2355–2366. 15519506 10.1016/j.ejca.2004.06.003

[2] Hollestein LM de Vries E Nijsten T Trends of cutaneous squamous cell carcinoma in the Netherlands: increased incidence rates, but stable relative survival and mortality 1989-2008. *Eur J Cancer* 2012; 48:2046–2053. 22342554 10.1016/j.ejca.2012.01.003

[3] Boyle P Autier P Bartelink H European code against cancer and scientific justification: third version (2003). *Ann Oncol* 2003; 14:973–1005. 12853336 10.1093/annonc/mdg305

[4] Gandini S Sera F Cattaruzza MS Meta-analysis of risk factors for cutaneous melanoma: II. Sun exposure. *Eur J Cancer* 2005; 41:45–60. 15617990 10.1016/j.ejca.2004.10.016

[5] El Ghissassi F Baan R Straif K A review of human carcinogens—part D: radiation. *Lancet Oncol* 2009; 10:751–752. 19655431 10.1016/s1470-2045(09)70213-x

[6] Lovatt TJ Lear JT Bastrilles J Associations between ultraviolet radiation, basal cell carcinoma site and histology, host characteristics, and rate of development of further tumors. *J Am Acad Dermatol* 2005; 52:468–473. 15761425 10.1016/j.jaad.2004.08.060

[7] Siskind V Whiteman DC Aitken JF An analysis of risk factors for cutaneous melanoma by anatomical site (Australia). *Cancer Causes Control* 2005; 16:193–199. 15947871 10.1007/s10552-004-4325-5

[8] Bleyer A O’Leary M Barr R Cancer epidemiology in older adolescents and young adults 15 to 29 years of age, including SEER incidence and survival: 1975–2000. 2006; Bethesda, MD: National Cancer Institute, NIH publication 06-5767.

[9] Reed KB Brewer JD Lohse CM Increasing incidence of melanoma among young adults: an epidemiological study in Olmsted County, Minnesota. *Mayo Clin Proc* 2012; 87:328–334. 22469345 10.1016/j.mayocp.2012.01.010 PMC3538462

[10] Christenson LJ Borrowman TA Vachon CM Incidence of basal cell and squamous cell carcinomas in a population younger than 40 years. *JAMA* 2005; 294:681–690. 16091570 10.1001/jama.294.6.681

[11] Coelho SG Hearing VJ UVA tanning is involved in the increased incidence of skin cancers in fair skinned young women. *Pigment Cell Melanoma Res* 2010; 23:57–63. 19968819 10.1111/j.1755-148X.2009.00656.x PMC2810005

[12] Boniol M Autier P Boyle P Cutaneous melanoma attributable to sunbed use: systematic review and meta-analysis. *BMJ* 2012; 345:e8503. 10.1136/bmj.e4757 PMC3404185 22833605

[13] Wehner MR Chren MM Nameth D International prevalence of indoor tanning: a systematic review and meta-analysis. *JAMA Dermatol* 2014; 150:390–400. 24477278 10.1001/jamadermatol.2013.6896 PMC4117411

[14] Italian Ministerial Decree of May 12, 2011 n. 110 published in the “Gazzetta Ufficiale” no. 163 of July 15. Regolamento di attuazione dell’articolo 10, comma 1, della legge 4 gennaio 1990, n. 1, relativo agli apparecchi elettromeccanici utilizzati per l’attivita’ di estetista. (2011). Available at: http://www/gazzettaufficiale.biz/atti/2011/20110163/011G0151.htm (accessed March 1, 2016).

[15] Pawlak MT Bui M Amir M Legislation restricting access to indoor tanning throughout the world. *Arch Dermatol* 2012; 148:1006–1012. 22801924 10.1001/archdermatol.2012.2080

[16] World Health Organization. INTERSUN. The global UV project: a guide and compendium. (2003). Available at: http://www.who.int/uv/publications/intersunguide/en/index.html (accessed March 1, 2016).

[17] World Health Organization. ENHIS fact sheet No. 4.8. Policies to reduce the excessive exposure of children to ultraviolet radiation. (2007). Available at: http://www.euro.who.int/_data/assets/pdf_file/0008/97451/4.8.pdf?ua=1 (accessed March 1, 2016).

[18] Saraiya M Glanz K Briss PA Interventions to prevent skin cancer by reducing exposure to ultraviolet radiation: a systematic review. *Am J Prev Med* 2004; 27:422–466. 15556744 10.1016/j.amepre.2004.08.009

[19] Stanganelli I Gandini S Magi S Sunbed use among subjects at high risk of melanoma: an Italian survey after the ban. *Br J Dermatol* 2013; 169:351–357. 23601037 10.1111/bjd.12384

[20] Gandini S Stanganelli I Magi S Melanoma attributable to sunbed use and tan seeking behaviours: an Italian survey. *Eur J Dermatol* 2014; 24:35–40. 24334101 10.1684/ejd.2013.2214

[21] Monfrecola G Fabbrocini G Posteraro G What do young people think about the dangers of sunbathing, skin cancer and sunbeds? A questionnaire survey among Italians. *Photodermatol Photoimmunol Photomed* 2000; 16:15–18. 10721859 10.1034/j.1600-0781.2000.160105.x

[22] Køster B Thorgaard C Philip A Sunbed use and campaign initiatives in the Danish population, 2007–2009: a cross-sectional study. *J Eur Acad Dermatol Venereol* 2011; 25:1351–1355. 21711466 10.1111/j.1468-3083.2010.03960.x

[23] Diehl K Bock C Greinert R Use of sunbeds by minors despite a legal regulation: extent, characteristics, and reasons. *J Public Health* 2013; 21:427–433.

[24] Guy GP Jr Tai E Richardson LC Use of indoor tanning devices by high school students in the United States, 2009. *Prev Chronic Dis* 2011; 8:A116. 21843419 PMC3181189

[25] Krarup AF Koster B Thorgaard C Sunbed use by children aged 8–18 years in Denmark in 2008: a cross-sectional study. *Br J Dermatol* 2011; 165:214–216. 21457215 10.1111/j.1365-2133.2011.10352.x

[26] Mayer JA Woodruff SI Slymen DJ Adolescents’ use of indoor tanning: a large-scale evaluation of psychosocial, environmental, and policy-level correlates. *Am J Public Health* 2011; 101:930–938. 21421947 10.2105/AJPH.2010.300079 PMC3076411

[27] Brandberg Y Ullen H Sjoberg L Sunbathing and sunbed use related to self-image in a randomized sample of Swedish adolescents. *Eur J Cancer Prev* 1998; 7:321–329. 9806121 10.1097/00008469-199808000-00008

[28] Aarestrup C Bonnesen CT Thygesen LC The effect of a school-based intervention on sunbed use in Danish pupils at continuation schools: a cluster randomized controlled trial. *J Adolesc Health* 2014; 54:214–220. 24119418 10.1016/j.jadohealth.2013.08.011

[29] Hill D Dixon H Promoting sun protection in children: rationale and challenges. *Health Educ Behav* 1999; 26:409–417. 10349577 10.1177/109019819902600310

[30] Dobbinson S Peipers A Reading D A national approach to skin cancer prevention: the National Sun Smart Schools Programme. *Med J Aust* 1998; 169:513–514. 9861906 10.5694/j.1326-5377.1998.tb123396.x

[31] Arthey S Clarke VA Sun tanning and sun protection: a review of the psychological literature. *Soc Sci Med* 1995; 40:265–274. 7899938 10.1016/0277-9536(94)e0063-x

[32] Lazovich D Forster J Sorensen G Characteristics associated with use or intention to use indoor tanning among adolescents. *Arch Pediatr Adolesc Med* 2004; 158:918–924. 15351760 10.1001/archpedi.158.9.918

[33] Cokkinides VE Weinstock MA O’Connell MC Use of indoor tanning sunlamps by US youth, ages 11–18 years, and by their parent or guardian caregivers: prevalence and correlates. *Pediatrics* 2002; 109:1124–1130. 12042553 10.1542/peds.109.6.1124

[34] Schneider S1 Zimmermann S Diehl K Sunbed use in German adults: risk awareness does not correlate with behaviour. *Acta Derm Venereol* 2009; 89:470–475. 19734971 10.2340/00015555-0689

[35] Holman DM Fox KA Glenn JD Strategies to reduce indoor tanning: current research gaps and future opportunities for prevention. *Am J Prev Med* 2013; 44:672–681. 23683986 10.1016/j.amepre.2013.02.014 PMC4413462

